# Jejunal Adenocarcinoma as a Rare Cause of Small Bowel Obstruction: A Case Report

**DOI:** 10.7759/cureus.10763

**Published:** 2020-10-02

**Authors:** Dua Azim, Sohail Kumar, Lajpat Rai, Khursheed Ahmed Samo, Amjad Siraj Memon

**Affiliations:** 1 Internal Medicine, Dow Medical College, Dr. Ruth K. M. Pfau Civil Hospital, Karachi, PAK; 2 Surgery, Dow Medical College, Dr. Ruth K. M. Pfau Civil Hospital, Karachi, PAK

**Keywords:** jejunal adenocarcinoma, small bowel adenocarcinoma, small bowel obstruction, stricture

## Abstract

Jejunal adenocarcinoma, a small bowel adenocarcinoma (SBA), is a rare cause of small bowel obstruction. Jejunal adenocarcinoma classically presents with vague clinical symptoms, i.e., abdominal pain, discomfort, and weight loss, making timely diagnosis challenging. Owing to its diagnosis at a late stage, the prognosis of jejunal adenocarcinoma is poor. Curative resection of the tumor at the early stages remains a treatment of choice. Here, we report a case of a 55-year-old man presenting with symptoms of nausea, vomiting, abdominal pain, abdominal distension, and relative constipation. Computed tomography (CT) scan showed dilated small bowel loops. Exploratory laparotomy was performed, which revealed a jejunal stricture and dilated small bowel loops proximal to it. Suspicious stricture, along with the diseased portion of the intestine, was removed through en-bloc resection. Histopathology and metastatic workup revealed moderately differentiated adenocarcinoma with stage IIB (T4aN0M0). We conclude that, although rare, jejunal adenocarcinoma should be kept in mind when dealing with a patient presenting with symptoms indicating small bowel obstruction. Our purpose is to emphasize laparotomy as both a diagnostic and surgical modality for SBAs in early stages, especially in setups of low economic countries where advanced imaging techniques are relatively inaccessible.

## Introduction

Small bowel obstruction is a surgical emergency that we encounter very frequently in our day-to-day practice. Postoperative adhesions (60%) are the most common cause of small bowel obstruction, followed by hernias, Crohn's disease, malignancy, and volvulus. Other less frequent causes of bowel obstruction differ between young adults and elderly patients; gallstone ileus is more frequent in elderly patients while Crohn’s disease in young adults [1]. Rarely small bowel obstruction may occur as a result of strictures secondary to small bowel adenocarcinoma (SBA) that accounts for about 3-5% of all gastrointestinal malignancies [2]. Duodenal adenocarcinoma is the most common (57%), whereas adenocarcinoma of the jejunum (29%) is still rare [3].

SBA usually presents in people aged between 55 and 65 years. It presents with vague symptoms such as abdominal discomfort, pain, nausea, vomiting, gastrointestinal bleeding, and intestinal obstruction. SBAs are often diagnosed in an emergency setting, either as an obstruction (40%) or bleeding (24%) [4]. The nonspecific presentation, together with the unavailability of any screening method, unfortunately, results in delayed diagnosis and an advanced stage of SBA on presentation. Complete resection provides the only means of cure in the early stages, and the role of adjuvant therapy remains uncertain [3]. There is a dearth of data on SBA, signifying that much is yet to be known.

Here, we present a case of a 55-year-old man with a small bowel obstruction caused by a primary jejunal adenocarcinoma.

## Case presentation

A 55-year-old man, with no known co-morbidities or any previous abdominal surgery, presented to the emergency department of Dr. Ruth K. M. Pfau, Civil Hospital, Karachi, with a two-week history of abdominal pain and distention, and a one-week history of constipation, nausea, and vomiting. He also reported a 10-kg weight loss in the past six months. Furthermore, he was kept on anti-tuberculosis therapy for six months when diagnosed with pulmonary tuberculosis two years ago. The patient's family history was insignificant.

**Figure 1:**
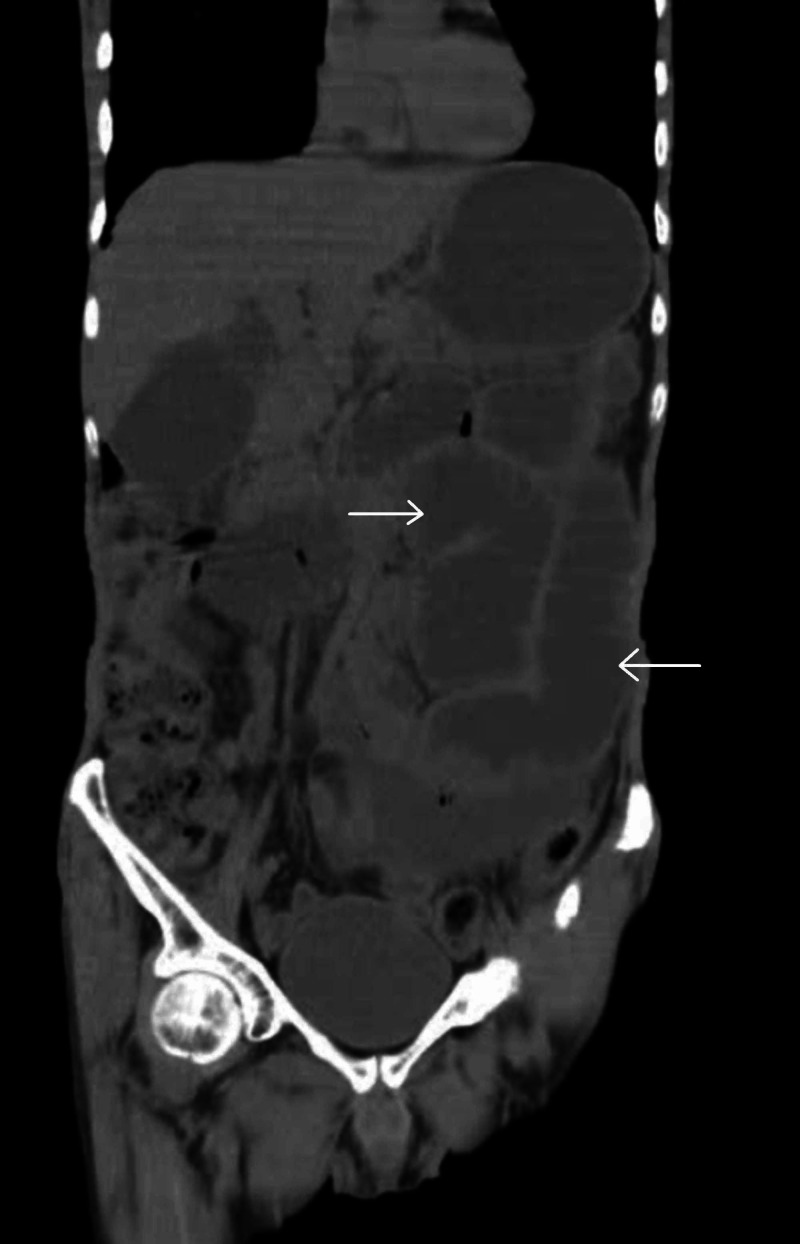
Preoperative coronal computed tomography (CT) scan of the abdomen showing dilated small bowel loops (arrows).

**Figure 2:**
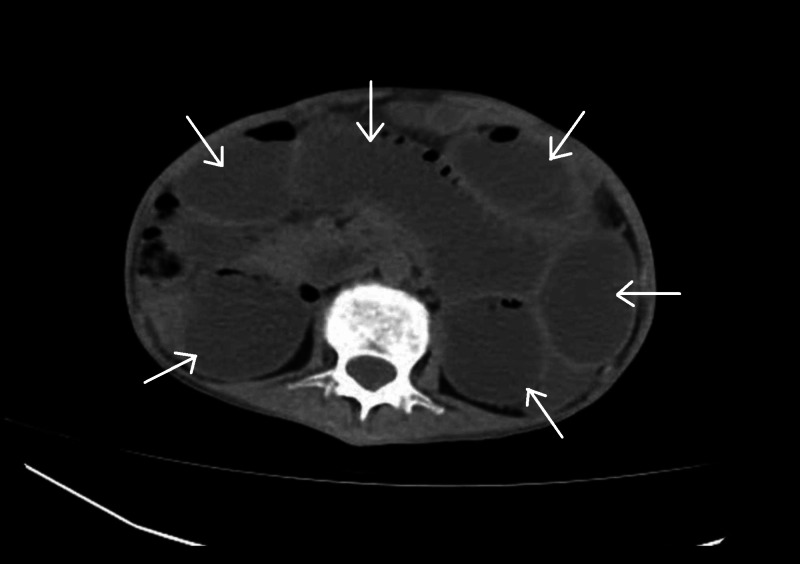
Preoperative axial computed tomography (CT) scan of the abdomen showing dilated small bowel loops (arrows).

On examination, vitals were normal; the patient was mildly dehydrated and resuscitated with ringer lactate. The abdomen was distended, with exaggerated bowel sounds. No mass was palpable on examination. The digital rectal examination (DRE) and the rest of the systemic examinations were unremarkable. Hematological investigations are demonstrated in Table 1. On radiological examination, computed tomography (CT) scan of the abdomen showed small bowel obstruction with dilated small bowel loops proximal to it, while the rest of the bowel was collapsed (Figures 1, 2). No discernable mass was noticed. Based on imaging, a preoperative diagnosis of small bowel mass with the differential diagnosis of small bowel malignancy/abdominal tuberculosis was made. Subsequently, an exploratory laparotomy was planned.

**Table 1:** Laboratory investigations of the patient. Hb: Hemoglobin; RBC: Red blood cells; Hct: Hematocrit; MCV: Mean corpuscular volume; MCH: Mean corpuscular hemoglobin; MCHC: Mean corpuscular hemoglobin concentration; TLC: Total leukocyte count; PLT: Platelet; CRP: C-reactive protein; BUN: Blood urea nitrogen; ALT: Alanine aminotransferase; ALP: Alkaline phosphatase.

Laboratory Investigation	Patient’s Result	Reference Range
Hb	13	13.0-18.0 g/dl
RBC	4.42	4.5-5.8 million/mcL
Hct	39.6	40-58%
MCV	89.6	76-96 fL
MCH	29.4	28-32 pg
MCHC	32.8	32-36 g/dL
TLC	11100	4,000-11,000/μL
Neutrophils	89	50-75%
Lymphocytes	6	20-50%
Monocytes	3	1-6%
Eosinophils	2	1-6%
Basophils	0	0-1%
PLT	312,000	150,000-400,000/μL
Sodium	139	136-146 mEq/L
Chloride	91	98-106 mEq/L
Potassium	3.9	3.5-5.1 mEq/L
CRP	102.6	<5 mg/L
BUN	20	6-20 mg/dL
Serum Creatinine	0.7	0.7-1.6 mg/dL
Bilirubin	0.47	<1.2 mg/dL
ALT	8	7-56 U/L
ALP	103	50-136 U/L

**Figure 3:**
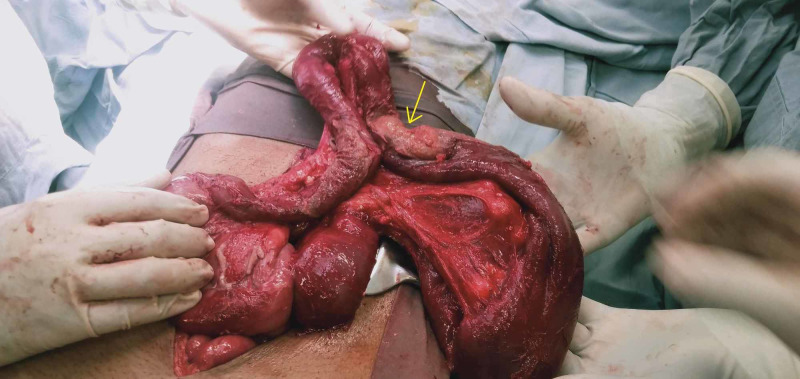
Intraoperative specimen showing suspicious growth stricture (arrow) at proximal jejunum and dilated small bowel loops proximal to it.

Intraoperative inspection showed stricture at the proximal jejunum. Adhesions in the whole abdomen and 200 ml of frank pus surrounding the stricture were observed. The small bowel was distended proximal to the stricture and collapsed distal to it (Figure 3). Intraoperative inspection of the abdominal cavity showed no evidence of metastatic lesions in the liver or peritoneum. En-bloc resection of the stricture with primary anastomosis was performed. Postoperative recovery was uneventful, and the patient was discharged after two days.

Histopathology confirmed a moderately differentiated adenocarcinoma of the jejunum, invading the serosa with tumor-free resection margins. On sectioning of the specimen, the mucosa of the bowel wall exhibited a lesion measuring 2 cm x 2 cm. We also recovered 27 lymph nodes; none of them was involved by the tumor. However, seven lymph nodes showed complete hyalinization. Lymphatic and vascular invasion was not identified. The cancer was, thus, staged as IIB (T4aN0M0). Postoperative CT scan of the chest, abdomen, and pelvis (CAP) showed no metastasis. Carcinoembryonic antigen (CEA) levels were 3.7 ng/ml (normal for a non-smoker, <3). Follow-up with a surveillance CT scan of CAP at six-month showed no recurrence of the disease. The patient remains well to date.

## Discussion

Although small bowel comprises 90% of the mucosal surface area and 75% of the length of the entire gastrointestinal tract, malignancies of the small bowel are relatively rare, accounting for about 3-5% of all gastrointestinal malignancies [2]. The most frequent location of SBA is the duodenum (57%), followed by jejunum (29%) and ileum (13%) [3]. The environment of the small bowel is anti-neoplastic; several features including, rapid transit of food, low bacterial content, presence of enzyme benzopyrene hydroxylase, rapid turnover of epithelial cells, IgA, and elaborated lymphoid tissue have protective effects against neoplastic growth [5].

SBAs are slightly more common in men than women and are commonly diagnosed at 60 years of age [4]. Risk factors for small bowel malignancies include smoking, alcohol consumption, preexisting adenoma, Crohn's disease, celiac sprue, hereditary nonpolyposis colorectal cancer, familial adenomatous polyposis, Peutz-Jeghers syndrome, and frequent consumption of red meat [2,6]. However, there were no such risk factors in the case discussed above.

SBA may present asymptomatically with microcytic anemia as the only sign of development [7]. However, patients usually present with complaints of vague clinical symptoms such as abdominal pain, discomfort, and weight loss, which makes a timely diagnosis challenging. Nausea/vomiting and bowel obstruction may also occur [8], as witnessed in our case. Furthermore, unusual presentations such as intestinal perforation and intussusception may require immediate surgery. Studies have shown that SBA is mainly diagnosed at advanced stages, such as stage III (having lymph node metastasis) and stage IV (having distant metastasis) in 40% and 35-40% of the cases, respectively [4,9]. However, our patient showed no lymph node and distant metastasis.

Various investigational modalities are available to detect small bowel tumors. Older imaging techniques have limited importance in the diagnosis of SBA. Barium meal follow-through has a sensitivity of 50% in the detection of the tumor whereas push enteroscopy can only assess up to 40% of the small bowel length [10]. According to studies, CT enteroclysis has been the most promising mode of investigation with a sensitivity of 100% [11]. Moreover, the advent of double-balloon enteroscopy and the development of wireless capsule endoscopy have made the diagnosis of small bowel adenocarcinoma unchallenging for a large number of cancer patients at a relatively early stage [12]. Since our patient presented in an emergency and was prepared for immediate surgery, we did not perform the aforementioned investigations.

Studies suggest that CT scan has an accuracy of 47% for the diagnosis of SBA and is a valuable mode of investigation for detecting local, regional, and distant metastasis; however, it may have value in diagnosing small primary lesions [2,13]. It is also evident in our case that while the CT scan hinted towards intestinal obstruction, it did not show any clear sign of a malignant lesion. Moreover, as described in our case, SBAs are majorly diagnosed intraoperatively and confirmed later on histopathology [14]. We also performed a CT scan of CAP to assess distant metastasis, as recommended by French guidelines [15]. The French guidelines highlight the importance of upper and lower gastrointestinal endoscopy to look for other tumors suggesting a predisposing genetic disease and serum tumor markers such as CEA and carbohydrate antigen (CA) 19-9 should be done at baseline, as they have prognostic value, especially in metastatic disease and recurrence.

The treatment of choice for SBA is complete en-bloc resection of the tumor as it offers the best overall survival. It involves the removal of the primary tumor with clear margins together with loco-regional lymph node resection. Thus, curative surgery offers a 50% chance of cure. Recurrence at distant sites is the predominant pattern of failure following a curative resection [8]. To date, no standard adjuvant regimen has been rendered truly effective against SBA [3,5].

In addition, patients of SBA have a poor prognosis with a five-year overall survival (OS) rate ranging from 14% to 33%. The five-year OS is related to the tumor stage, with stage IV tumor having the worst prognosis (3-5%). Moreover, the male gender, age greater than 55 years, lymph node invasion, and distant metastasis also contribute to a poor outcome. Several studies suggest that a primary duodenal tumor has a worse prognosis than a primary jejunal or ileal tumor [4,9].

We aim to report a case of adenocarcinoma of the jejunum as a rare cause of small bowel obstruction and emphasize the importance of laparotomy in the management of patients with small bowel obstruction, where CT findings are inconclusive. If SBA is suspected based on operative findings, cancer-directed surgery proves to be the most appropriate approach to its best survival outcome. A surgeon should be wise enough to make timely decisions, thereby preventing a delay in diagnosis and subsequent poorer outcomes of aggressive disease.

## Conclusions

Jejunal adenocarcinoma, a small bowel adenocarcinoma, is a rare cause of small bowel obstruction. Patients usually present with vague clinical features such as abdominal discomfort, pain, and weight loss, which makes the timely diagnosis challenging. Although rare, jejunal adenocarcinoma should be kept in mind when dealing with a patient presenting with symptoms indicating small bowel obstruction. Laparotomy may serve as both diagnostic and surgical modality for SBAs in early stages, especially in setups of low economic countries where the facilities of advanced imaging techniques are not readily available. Further, profound research is required to assess the potential applications of newer investigative strategies and treatment modalities to achieve better disease outcomes.
